# Continuous crossbreeding of sake yeasts using growth selection systems for a-type and α-type cells

**DOI:** 10.1186/s13568-016-0216-x

**Published:** 2016-07-08

**Authors:** Nobuo Fukuda, Misato Kaishima, Jun Ishii, Akihiko Kondo, Shinya Honda

**Affiliations:** Biomedical Research Institute, National Institute of Advanced Industrial Science and Technology (AIST), Higashi, Tsukuba, Ibaraki 305-8566 Japan; Department of Chemical Science and Engineering, Graduate School of Engineering, Kobe University, 1-1 Rokkodai, Nada, Kobe, Japan; Organization of Advanced Science and Technology, Kobe University, 1-1 Rokkodai, Nada, Kobe, Japan

**Keywords:** Sake yeast, Crossbreeding, Mating type, Growth selection, Hybrid strain

## Abstract

Sake yeasts belong to the budding yeast species *Saccharomyces cerevisiae* and have high fermentation activity and ethanol production. Although the traditional crossbreeding of sake yeasts is a time-consuming and inefficient process due to the low sporulation rates and spore viability of these strains, considerable effort has been devoted to the development of hybrid strains with superior brewing characteristics. In the present work, we describe a growth selection system for **a**- and α-type cells aimed at the crossbreeding of industrial yeasts, and performed hybridizations with sake yeast strains Kyokai No. 6, No. 7 and No. 9 to examine the feasibility of this approach. We successfully generated both **a**- and α-type strains from all parental strains, and acquired six types of hybrids by outcrossing. One of these hybrid strains was subjected to continuous crossbreeding, yielding the multi-hybrid strain, which inherited the genetic characteristics of Kyokai No. 6, No. 7 and No. 9. Notably, because all of the genetic modifications of the yeast cells were introduced using plasmids, these traits can be easily removed. The approach described here has the potential to markedly accelerate the crossbreeding of industrial yeast strains with desirable properties.

**Electronic supplementary material:**

The online version of this article (doi:10.1186/s13568-016-0216-x) contains supplementary material, which is available to authorized users.

## Introduction

Sake is a traditional Japanese alcoholic beverage made from fermented rice. In the production of sake, rice starch is first degraded by the koji fungus *Aspergillus oryzae* into glucose, which is then fermented to ethanol by sake yeast, strains of the budding yeast species *Saccharomyces cerevisiae* (Kitagaki and Kitamoto 2013; Shiroma et al. 2014). Sake yeasts have many characteristics suitable for sake brewing, such as aromatic production and high ethanol tolerance (Katou et al. 2008). Sake yeast strains have been selected through several hundred years of brewing (Shiroma et al. 2014); however, more rapid methods for generating new and superior strains are highly desirable.

Crossbreeding is an attractive approach to improve and combine traits of different yeast strains (Higgins et al. 2001; Kishimoto 1994; Shinohara et al. 1997). Common breeding strategies, such as backcrossing and multi-hybridization, require cycles of hybridization (continuous crossbreeding). Most sake yeasts are *MAT****a****/α* diploid strains and are unable to mate directly; therefore, the isolation of *MAT****a*** and *MATα* haploid strains via sporulation is a prerequisite for crossbreeding. However, because industrially used sake yeast strains, such as Kyokai No. 7 and No. 9, have low sporulation rates (Suizu et al. 1996), the crossbreeding of sake yeast strains is inefficient and technically challenging.

To overcome the problem of poor sporulation, it is possible to select for strains that have undergone spontaneous chromosomal aberrations, such as loss of heterozygosity (LOH) and mitotic chromosome loss, during mitotic division to obtain **a**- and α-type yeast cells that possesses similar mating abilities as *MAT****a*** and *MATα* haploids generated via sporulation (Fukuda et al. 2013a). LOH is a natural genetic event that generates homozygous loci via chromosomal rearrangement of heterozygous loci (Alvaro et al. 2006; Andersen et al. 2008; Daigaku et al. 2004; Takagi et al. 2008), whereas mitotic chromosome loss, which is also a naturally occurring event, involves the loss of single or multiple chromosomes (Mayer and Aguilera 1990). Therefore, a LOH event at the mating-type (*MAT*) locus within *MAT****a****/α* cells produces either *MAT****a****/****a*** or *MATα/α* cells, whereas yeast cells that lose one of both copies of chromosome III containing the *MAT* locus during mitotic division become *MAT****a*** or *MATα* cells (Fukuda et al. 2013a). However, because the spontaneous occurrence frequencies of LOH and mitotic chromosome loss are less than 1 × 10^−4^, it is difficult to isolate the generated **a**- and α-type cells from mixed cell populations (Hiraoka et al. 2000; Kumaran et al. 2013).

To isolate **a**- and α-type cells, we previously established a growth selection system for laboratory yeast strains using the auxotrophic marker *URA3* (Fukuda et al. 2013a). In this system, the expression of the marker gene is induced in a mating-type-specific manner, thereby permitting the efficient selection of **a**- and α-cells from within a mixed cell population. Using this approach, we succeeded in isolating **a**- and α-type derivative cells from a cell population of parental *MAT****a****/α* laboratory yeasts without any false positives, and confirmed that these cells were able to mate and produce new hybrid yeasts.

Unlike auxotrophic laboratory yeasts, however, industrially used yeasts, including sake yeasts, are generally prototrophic, which prevents the use of auxotrophic markers for the selection of strains following mating. In addition, industrial yeast strains have remarkably low genetic transformation efficiencies compared to laboratory strains (sake yeast, 10^1^~10^2^ cfu/μg-DNA; laboratory yeast, 10^4^~10^5^ cfu/μg-DNA) (Ogata et al. 1993). Therefore, a complete method for the transformation, isolation, and evaluation of **a**- and α-type derivative and hybrid cells, is required for the efficient crossbreeding of sake yeasts.

**Fig. 1:**
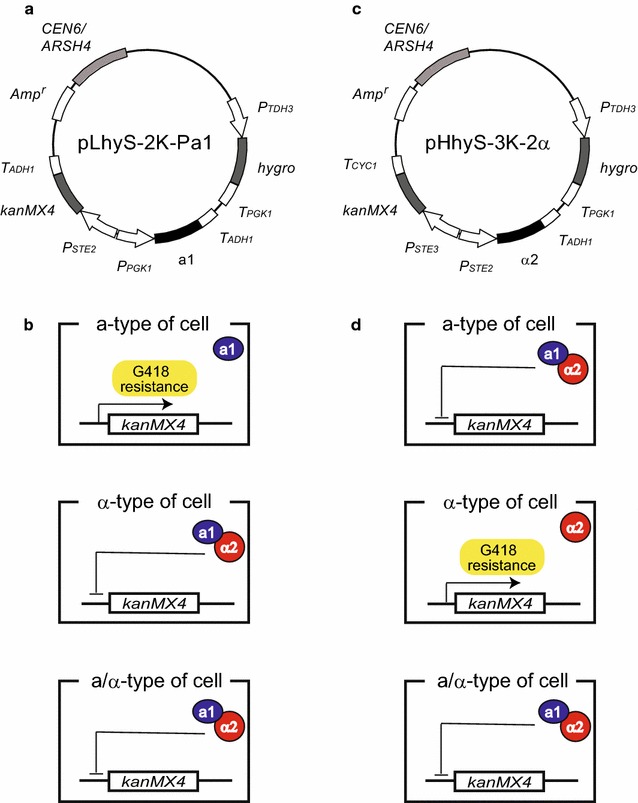
Schematic outline of the strategy used for isolation of a- and α-type yeast cells. **a** Plasmid map of pLhyS-2 K-Pa1, which was used for the isolation of **a**-type yeast cells. **b** Isolation of **a**-type yeast cells using G418 selection. Formation of the a1-α2 complex in α-type and **a**/α-type cells represses expression of the *kanMX4* marker gene. Only **a**-type cells are able to survive in culture medium containing G418 by expressing the *kanMX4* marker gene. **c** Plasmid map of pHhyS-3 K-2α, which was used for the isolation of α-type yeast cells. **d** When introduced into **a**-type and **a**/α-type cells, the α2 protein represses expression of the *kanMX4* marker gene. Only α-type cells are able to survive in culture medium containing G418 by expressing the *kanMX4* marker gene

Here, we designed and constructed two types of plasmids for isolating **a**- and α-type sake yeasts from mixed cell populations (Fig. 1). Although drug sensitivity varies from strain to strain, the *hygro* (hygromycin B-resistance) and *kanMX4* genes (G418-resistance) have been commonly used for the selection of prototrophic yeasts (Murakami et al. 2012). To develop a versatile method for crossbreeding of sake yeasts, we used the *hygro* gene as a transformation marker and the *kanMX4* gene as a marker for isolation of **a**- or α-type derivative cells. In this approach, the **a**- and α-type derivative cells must have different marker genes. Because yeast transformation is performed using plasmids in our system, the marker genes are lost in the absence of selection pressure. Therefore, through plasmid exchange, different marker genes for hybridization can be introduced into the target **a**- and α-type derivative cells by removing unnecessary plasmids and introducing new plasmids. In this work, the feasibility of this approach for sake yeasts was demonstrated by performing and generating modified strains through continuous crossbreeding.

## Materials and methods

### Strains and media

Detailed information about *S. cerevisiae* laboratory yeast strain BY4742 (Brachmann et al. 1998) and sake yeast strains Kyokai No. 6, No. 7 and No. 9 (provided by the Biological Resource Center, NITE, Japan), as well as the other strains used in this study, is shown in Table 1. Yeast cells were grown in YPD medium (1 % yeast extract, 2 % peptone and 2 % glucose) or SD/MSG medium (0.17 % yeast nitrogen base without amino acids and ammonium sulfate [Becton–Dickinson and Company, Franklin Lakes, NJ, USA], 0.1 % monosodium glutamate and 2 % glucose). A final concentration of 2 % agar was added to both types of media to prepare solid media.

**Table 1:** Yeast strains and plasmids used in this study

Name description	Description	Reference source
*Yeast strains*		
BY4742	*MATα* *his3∆1 ura3∆0 leu2∆0 lys2∆0*	Brachmann et al. (1998)
MCF4741	*MAT* ***a*** *his3∆1 ura3∆0 leu2∆0 met15∆0 Fig.* *1::Fig.* *1*-*EGFP*-*loxP*-*kanMX4*-*loxP*	Fukuda et al. (2013b)
HR42-11T	*MATα his3∆1 ura3∆0 leu2∆0 lys2∆0 hmr* ***a***_*inc*_ *::HMR* ***a***_*cut*_-*loxP*-*kanMX4*-*loxP*	Fukuda et al. (2013b)
K6	Sake yeast; Kyokai No. 6; *MAT* ***a/*** *α*	NBRC2346^a^
K7	Sake yeast; Kyokai No. 7; *MAT* ***a/*** *α*	NBRC2347^a^
K9	Sake yeast; Kyokai No. 9; *MAT* ***a/*** *α*	NBRC2377^a^
K6A	**a**-type of strain derived from Kyokai No. 6	Present study
K6AL	α-type of strain derived from Kyokai No. 6	Present study
K7A	**a**-type of strain derived from Kyokai No. 7	Present study
K7AL	α-type of strain derived from Kyokai No. 7	Present study
K9A	**a**-type of strain derived from Kyokai No. 9	Present study
K9AL	α-type of strain derived from Kyokai No. 9	Present study
K67	Hybrid strain generated by zygosis of K6A and K7AL	Present study
K69	Hybrid strain generated by zygosis of K6A and K9AL	Present study
K76	Hybrid strain generated by zygosis of K7A and K6AL	Present study
K79	Hybrid strain generated by zygosis of K7A and K9AL	Present study
K96	Hybrid strain generated by zygosis of K9A and K6AL	Present study
K97	Hybrid strain generated by zygosis of K9A and K7AL	Present study
K76A	**a**-type of strain derived from K76	Present study
K76AL	α-type of strain derived from K76	Present study
K76x9	Hybrid strain generated by zygosis of K76A and K9AL	Present study
*Plasmids*		
pLY-hygro	*2*μ ori, *LEU2* marker and *P*_*TDH3*_-*hygro*	Present study
pHY-kan	*2*μ ori, *HIS3* marker and *P*_*TEF1*_-*kanMX4*	Present study
pAUR112	*CEN4/ARS1* ori, *URA3*, *AUR1*-*C*	Takara Bio, Inc., Shiga, Japan
pLhyS-2 K-Pa1	*CEN6/ARSH4* ori, *LEU2*, *P*_*TDH3*_-*hygro, P*_*STE2*_-*kanMX*4 and *P*_*PGK1*_-*a1*	Present study
pHhyS-3 K-2α	*CEN6/ARSH4* ori, *HIS3*, *P*_*TDH3*_-*hygro, P*_*STE3*_-*kanMX*4 and *P*_*STE2*_-*α2*	Present study

^a^Resources were provided by Biological Resource Center (NBRC), NITE, Japan

### Construction of plasmids

The sequences of the oligonucleotides used in this study are listed in Table 2. The plasmids used in this study (Table 1) were constructed as follows. Using pTriEx-2 Hygro (Novagen, Inc., Madison, WI) as a template, the *hygro* gene was amplified with oligonucleotide pair o1 and o2. *P*_*TDH3*_ was also amplified from genomic DNA derived from strain BY4742 using oligonucleotide pair o3 and o4. The two amplified DNA fragments, *P*_*TDH3*_ and *hygro*, were combined using the In-Fusion HD Cloning kit (Takara Bio, Inc., Shiga, Japan), and the resulting DNA fragment, *P*_*TDH3*_-*hygro*, was amplified with oligonucleotide pair o3 and o2, and then digested with *Sac*I and *Bam*HI. In addition, *T*_*PGK1*_ was amplified from genomic DNA derived from strain BY4742 using oligonucleotide pair o5 and o6, and the obtained fragment was digested with *Bam*HI and *Xho*I. The two digested DNA fragments, *P*_*TDH3*_-*hygro* and *T*_*PGK1*_, were inserted at the *Sac*I-*Xho*I sites of pLY-3U to replace the *P*_*STE3*_-*URA3*-*T*_*CYC1*_ cassette (Fukuda et al. 2013a), yielding a plasmid designated pLY-hygro.

**Table 2:** Sequences of oligonucleotides used to construct plasmids

Number	Sequence
1	5′-CAAAgcggccgcATGGATAGATCCGGAAAGCC-3′
2	5′-AATTTATTTCggatccCTATTCCTTTGCCCTCGGAC-3′
3	5′-TATAGGGCGAATTGgagctcGAATAAAAAACACGCTTTTT-3′
4	5′-CATgcggccgcTTTGTTTGTTTATGTGTGTT-3′
5	5′-GCTTATGTAAggatccGAAATAAATTGAATTGAAT-3′
6	5′-CGGGCCCCCCctcgagAGCTTTAACGAACGCAGAA-3′
7	5′-AATTGGAGCTCCAccgcggATCTGTTTAGCTTGCCTCGT-3′
8	5′-CGGGCCCCCCctcgagCTCGTTTTCGACACTGGAT-3′
9	5′-CTCGAGGGGGGGCCCGgagctcAGCTTTAACGAACGCAGAA-3′
10	5′-GGTGATATTGGATaccgcggAGATGCCGATTTGGGC-3′
11	5′-CGGGCCCCCCctcgag-3′
12	5′-TTTTCAACAAAATccgcgg-3′
13	5′-CGGGCCCCCCctcgagGAGCGACCTCATGCTATA-3′

Using pK6 (Fukuda et al. 2013b) as a template, the expression cassette of the *kanMX4* marker (consisting of *P*_*TEF1*_, ORF and terminator) was amplified with oligonucleotide pair o7 and o8, and inserted in place of the *P*_*PGK1*_-*EGFP*-*T*_*ADH1*_ cassette at the *Sac*II-*Xho*I sites of pHY-PGA (Fukuda et al. 2013a), yielding a plasmid designated pHY-kan.

Using pLY-hygro as a template, a DNA fragment containing the *P*_*TDH3*_-*hygro*-*T*_*PGK1*_ cassette was amplified with oligonucleotide pair o3 and o9. Subsequently, a DNA fragment containing the *P*_*PGK1*_-*a1*-*T*_*ADH1*_ cassette was amplified with oligonucleotide pair o10 and o11 from pHPY-a1 (Fukuda et al. 2013b). Using the In-Fusion kit, the two amplified DNA fragments, *P*_*TDH3*_-*hygro*-*T*_*PGK1*_ and *P*_*PGK1*_-*a1*-*T*_*ADH1*_, were inserted into the *Sac*I-*Sac*II sites of pLS-2 K (Fukuda et al. 2013a), yielding a plasmid designated pLhyS-2 K-Pa1.

Similarly, a DNA fragment containing the *P*_*STE2*_-*α2*-*T*_*ADH1*_ cassette was amplified with oligonucleotide pair o12 and o13 from pL3G-2α (Fukuda et al. 2013b). Using the In-Fusion kit, the two DNA fragments *P*_*TDH3*_-*hygro*-*T*_*PGK1*_ and *P*_*STE2*_-*α2*-*T*_*ADH1*_ were inserted into the *Sac*I-*Sac*II sites of pHS-3 K (Fukuda et al. 2013a), yielding a plasmid designated pHhyS-3 K-2α.

Each constructed plasmid was introduced into yeast cells using the lithium acetate method (Gietz et al. 1992).

### Investigation of cell growth characteristics

Each yeast strain was grown at 30 °C in 500 μL YPD medium supplemented with or without the antibiotics hygromycin B (HYG), geneticin (G418) or aureobasidin A (AUR; Takara Bio, Inc., Shiga, Japan) at the indicated concentrations. The initial optical density at 600 nm (OD_600_) values of the cultures were 0.03, and changes in the OD_600_ were monitored using a UV/visible spectrophotometer (Ultrospec 3100 pro; GE Healthcare Japan Corp., Tokyo, Japan).

### Isolation of yeast cells with target mating-type

Parental **a**/α-type cells were grown in 500 μL YPD medium containing 500 μg/mL HYG at 30 °C for 2 days, and were passaged daily with 10,000-fold dilution. Yeast cells were then harvested and washed and resuspended in distilled water. Cell suspensions were spread on YPD + G418 (500 μg/mL for the isolation of **a**-type cells, and 1.0 mg/mL for α-type cells) plates to isolate **a**- and α-type yeast cells.

### Mating assay

Evaluation of mating ability was performed by cultivating yeast cells with a mating partner in 1 ml YPD medium at 30 °C for 1.5 h. The initial OD_600_ of each strain was 0.1. After cultivation, yeast cells were harvested, washed, and resuspended in distilled water to give cell suspensions with OD_600_ values of 1, 0.1, and 0.01. A total of 10 μl of each cell suspensions was spotted on SD/MSG solid medium (without amino acids) containing 500 μg/mL G418 for the growth selection of zygotes. After incubation of the plates at 30 °C for 2 days, image data was recorded for colonies that formed on the solid medium.

### Ploidy analysis using FACS

Each yeast strain was grown overnight in 500 μL YPD medium at 30 °C. The cells then were harvested, washed with 500 μL distilled water, and resuspended in 500 μL of 70 % ethanol. After a 1-h incubation at room temperature, the ethanol-treated cells were harvested, washed with 500 μL phosphate buffered saline (PBS), and resuspended in 90 μL PBS containing 0.5 mg/mL RNase A. After 1 h of incubation at 37 °C, 10 μL of 1 mg/mL propidium iodide (PI) solution was added to the cell suspension, which was further incubated at 37 °C for 30 min to stain DNA. The stained cells were harvested, washed with 100 μL PBS, and resuspended in 1 mL sheath solution (Becton, Dickinson and Co., Franklin Lakes, NJ, USA). After a 5-s sonication to reduce cell flocculation, PI-fluorescence of yeast cells was detected using a BD FACS Canto II flow cytometer (Becton, Dickinson and Co., Franklin Lakes, NJ, USA) equipped with a 488-nm blue laser, and the collected data were an analyzed using FlowJo software (Tree star, Ashland, Oregon, USA). The fluorescence signal was collected through a 585/21 nm band-pass filter.

## Results

### General isolation strategy for a- and α-type yeast cells

An outline of the strategy used for the isolation of **a**- and α-type sake yeast cells is shown in Fig. 1. Typically, **a**-type cells express the a1 gene, whereas α-type haploids express the α2 gene from their respective *MAT* loci. Haploid-specific genes (*hsg*) are expressed and induce mating responses in both cell types. In contrast, **a**/α-type cells express both the a1 and α2 genes from *MAT* loci, resulting in the formation of the a1-α2 complex, which represses the expression of *hsg* (Fukuda et al. 2013b). Here, using machinery for mating-type-dependent gene expression (Fukuda et al. 2013a), the *kanMX4* marker gene was expressed in **a**- and α-type derivative cells.

The plasmid pLhyS-2 K-Pa1 (Fig. 1a) was constructed for the selection of **a**-type yeast cells and contains the *hygro* gene as a transformation marker (hygromycin B-resistance), the a1 gene for preventing undesirable mating between derivatives from the same parent by formation of the a1-α2 complex (Fukuda et al. 2013a, b), and the *kanMX4* marker gene (repressed by α2 alone) (Fig. 1b). For the selection of α-type yeast cells, the plasmid pHhyS-3 K-2α (Fig. 1c), which contains the *hygro* gene as a transformation marker, the α2 gene for the preventing undesirable mating by formation of the a1-α2 complex, and the *kanMX4* marker gene (repressed by the a1-α2 complex), was constructed (Fig. 1d). More details of the gene expression regulation system used in these constructs are described in previous reports (Fukuda et al. 2013a, b).

**Fig. 2:**
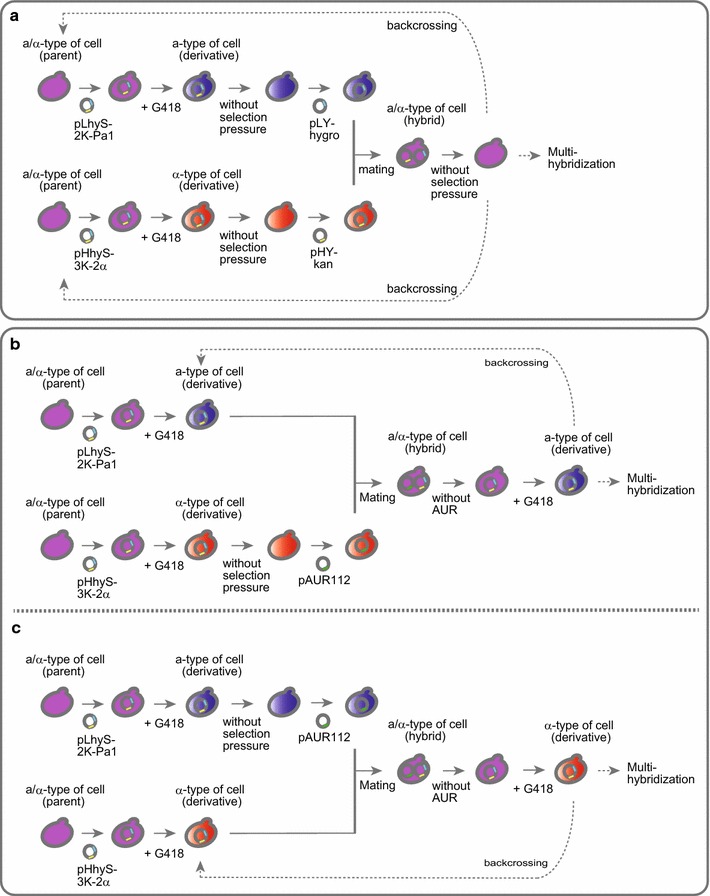
Schematic outline of the experimental design for the crossbreeding of sake yeast strains. **a** Outline of the basic method used for the crossbreeding of sake yeast strains. Plasmids pLhyS-2 K-Pa1 and pHhyS-3 K-2α were used for isolation of **a**- and α-type derivative cells, respectively, and plasmids pLY-hygro and pHY-kan were used for the hybridization of derivatives. Unnecessary plasmids were removed from yeast cells by cultivating cells in the absence of selection pressure. **b** Improved method for continuous crossbreeding without the requirement for removal of plasmid pLhyS-2 K-Pa1. **c** A second improved method for continuous crossbreeding without the requirement for removal of plasmid pHhyS-3 K-2α. Plasmid pAUR112 was used for the hybridization of obtained derivatives in (**b**) and (**c**)

The basic experimental design for the generation of **a**- and α-type sake yeast cells is schematically illustrated in Fig. 2a. Briefly, the plasmid pLhyS-2 K-Pa1 was introduced into parental **a**/α-type sake yeast strains to isolate **a**-type of derivatives, whereas the plasmid pHhyS-3 K-2α was used to transform other **a**/α-type strains to isolate α-type derivatives. In both cases, positive transformants were selected on solid medium containing HYG (YPD + HYG plates). After several passages of the transformant cultures, each cell mixture was individually spread on solid medium containing G418 (YPD + G418 plates) for the selection of **a**- or α-type cells expressing the *kanMX4* gene.

Hybrid cells cannot be generated via the mating of derivative **a**- and α-type cells, because both cell types express the same marker genes (*hygro* and *kanMX4*). To allow differentiation of the **a**- and α-type cells, plasmid exchanges were performed to introduce unique selection maker gene (Fig. 2a). The plasmids pLhyS-2 K-Pa1 and pHhyS-3 K-2α were removed by passaging cells in the absence of selection pressure, and the plasmids pLY-hygro (HYG-resistance) and pHY-kan (G418-resistance) were then introduced into derivative **a**-type and α-type cells, respectively. Hybrid **a**/α-type cells were selected on solid YPD medium containing HYG and G418 (YPD + HYG, G418 plates) and were then isolated on YPD plates after the removal of both plasmids through passage cultures performed with YPD medium without antibiotics.

Although repetitive hybridizations using the newly generated **a**/α-type sake yeast strains as the parent (continuous crossbreeding) can be performed using the scheme described in Fig. 2a, it is necessary to re-transform the strains with pLhyS-2 K-Pa1 or pHhyS-3 K-2α. To avoid the labor required for plasmid exchange for continuous crossbreeding, a third selection marker gene, *AUR1*-*C*, was used to generate **a**/α-type hybrid strains (Fig. 2b, c).

Figure 2b shows the procedure used to acquire **a**-type cells derived from the generated hybrids. After isolation of the derivatives from the parent **a**/α-strains, α-type cells were cultivated without selection pressure to remove the plasmid pHhyS-3 K-2α, and plasmid pAUR112 (Takara Bio, Inc., Shiga, Japan), which contains the *AUR1*-*C* marker gene, was then introduced into the α-type derivative cells. The mating of **a**- and α-type derivative cells was performed in mixed cultures in YPD medium, and **a**/α-type hybrid cells were the isolated on solid YPD medium containing HYG and AUR (YPD + HYG, AUR plates). After the removal of pAUR112 from cells by repeated passage in medium containing only HYG (YPD + HYG medium), **a**-type derivative cells expressing the *kanMX4* gene were selected on YPD + G418 plates. The isolated **a**-type cells can be directly utilized for hybridization, such as backcrossing and multi-hybridization, without removing the plasmid pLhyS-2 K-Pa1.

Figure 2c illustrates the procedure used to acquire **α**-type cells from the **a**/α-type hybrids. After isolation of the derivatives from the parent **a**/α-type of strains, **a**-type cells were cultivated without selection pressure to remove the plasmid pLhyS-2 K-Pa1, and plasmid pAUR112 was then introduced into the **a**-type derivative cells. The **a**- and α-type derivative cells were mated in YPD medium, and **a**/α-type hybrid cells were then isolated on YPD + HYG, AUR plates. After removal of pAUR112 by repeated passage in YPD + HYG medium, **a**-type cells expressing the *kanMX4* gene were selected on YPD + G418 plates. As described for the **a**-type cells generated using this procedure, the plasmid pHhyS-3 K-2α does not need to be removed from the isolated α-type cells prior to performing subsequent hybridizations.

### Outcrossing of Kyokai No. 6, No. 7 and No. 9

The basic method for generating strains for continuous crossbreeding (Fig. 2a) was utilized for the outcrossing of the sake yeast strains Kyokai No. 6, No. 7 and No. 9. The minimum inhibitory concentration (MIC) of HYG and G418 was first determined for these three strains (Additional file 1: Table S1 and S2). The MIC values of HYG for strains Kyokai No. 6, No. 7 and No. 9 were 300, 200 and 300 μg/ml, respectively, and the MIC of G418 was 200 μg/ml for all three strains.

To isolate **a**- and α-type cells of Kyokai No. 6, No. 7 and No. 9, cells transformed with pLhyS-2 K-Pa1 or pHhyS-3 K-2α were selected on YPD plates containing 300 μg/ml HYG, and after repeated passaging, cell suspensions of each transformant were spread on YPD + G418 (>500 μg/mL) plates. The plasmids pLhyS-2 K-Pa1 and pHhyS-3 K-2α were removed from the isolated **a**- or α-type yeast cells, as shown in Fig. 2a, yielding strains K6A, K7A and K9A (**a**-type), and K6AL, K7AL and K9AL (α-type) (Table 1). The removal of the plasmids containing the *hygro* and *kanMX4* genes was confirmed by PCR (Additional file 1: Table S3 and Fig. S1).

**Fig. 3:**
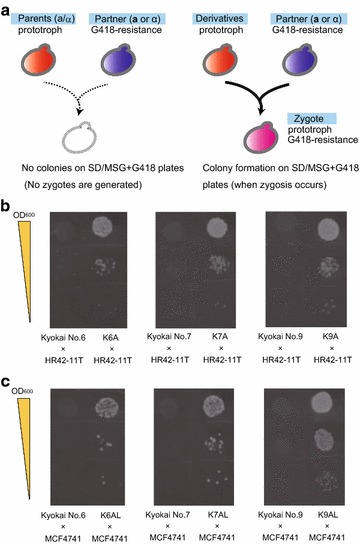
Evaluation of the mating abilities of derivative strains. **a** Schematic outline of the mating assay. Using haploid strains HR42-11T (α-cell) and MC4741 (**a**-cell) as the mating partners, zygotes were selected on solid medium containing G418, but lacking amino acids (SD/MSG + G418 plates). The Parent strains were Kyokai No. 6, No. 7 and No. 9 (**a**/α-type cells), and the Derivative strains were K6A, K7A and K9A (isolated as **a**-type cells) and K6AL, K7AL and K9AL (isolated as α-type cells), respectively. **b** Images showing colony formation in the mating assay for strains K6A, K7A and K9A. **c** Images showing colony formation in the mating assay for strains K6AL, K7AL and K9AL. The OD_600_ values of 10-μL cell suspensions spotted on the solid media were set at 1.0, 0.1 and 0.01

To verify the mating type and examine the mating abilities of the derivative strains, a mating assay was performed with the auxotrophic and G418-resistant laboratory haploid strains HR42-11T (α-type) and MCF4741 (**a**-type) (Fukuda et al. 2013b) (Table 1). Although all of the derivative strains are prototrophs, they do not have resistance to G418, and therefore only zygotes can grow on SD/MSG plates containing G418, but lacking any amino acids (Fig. 3a). In the mating assay, the derivative strains K6A, K7A and K9A mated with HR42-11T (α-type), whereas the parental strains (Kyokai No. 6, No. 7 and No. 9) did not mate (Fig. 3b). Similarly, the derivative strains K6AL, K7AL and K9AL mated with MCF4741 (**a**-type), whereas the parental strains again did not mate (Fig. 3c). These results confirmed that the growth selection system designed here promoted the generation and allowed for the isolation of **a-** and α-type yeast derivative cells.

Outcrossing of Kyokai No. 6, No. 7 and No. 9 was next conducted according to the scheme illustrated in Fig. 2a. After transformation of **a**- and α-type derivative cells with the plasmids pLY-hygro and pHY-kan, cells were selected on YPD + HYG and YPD + G418 plates, respectively. Zygotes (hybrid cells) were isolated from mixed cultures of the derivative cells using YPD + HYG, G418 plates, and all plasmids were then removed from each hybrid cell, yielding hybrid strains K67, K69, K76, K79, K96 and K97 (Table 1).

### Improvement of the continuous crossbreeding procedure

Although the feasibility of the basic hybrid selection method (Fig. 2a) was demonstrated in the above-described outcrossing experiment, each stage of the continuous crossbreeding procedure requires the exchange of plasmids. As plasmid exchange is a time- and labor-consuming process, we attempted to improve the continuous crossbreeding method (Fig. 2b, c) using a third marker gene (*AUR1*-*C*; AUR-resistance). Using this approach, **a-** and α-type derivative cells were isolated from hybrid strain K76.

In this modified selection system, the isolation of **a-** and α-type derivative cells based on transformation with the plasmids pLhyS-2 K-Pa1 and pHhyS-3 K-2α was performed as described in Fig. 2a. The obtained derivative cells, K7A/pLhyS-2 K-Pa1 and K6AL/pHhyS-3 K-2α, were then subjected to plasmid exchange using one of two methods. Because the MIC values of AUR against strains K7A and K6AL were 100 and 300 ng/ml, respectively (Additional file 1: Table S4), yeast transformation with plasmid pAUR112 was performed using YPD plates containing 300 ng/ml AUR.

To obtain **a**-type derivative cells from hybrid strain K76 according to the scheme illustrated in Fig. 2b, plasmid pAUR112 was introduced into strain K6AL after removing plasmid pHhyS-3 K-2α, yielding transformants K6AL/pAUR112 on YPD + AUR plates. K6AL/pAUR112 cells were cultured together with K7A/pLhyS-2 K-Pa1 cells, and hybrid K76/pLhyS-2 K-Pa1/pAUR112 cells were then isolated on YPD + HYG, AUR plates and further cultivated in YPD + HYG medium (without AUR). The cells were transferred to YPD + G418 plates for the selection of **a**-type derivative cells (K76A/pLhyS-2 K-Pa1). To obtain α-type derivative cells from hybrid strain K76 according to the scheme illustrated in Fig. 2c, plasmid pAUR112 was introduced into strain K7A after removing plasmid pLhyS-2 K-Pa1, yielding K7A/pAUR112 transformants, which were selected on YPD + AUR plates. Hybrid K76/pHhyS-3 K-2α/pAUR112 cells were then isolated on YPD + HYG, AUR plates from mixed cultures of K7A/pAUR112 and K6AL/pHhyS-3 K-2α cells, and were further cultivated in YPD + HYG medium (without AUR). Finally, **a**-type derivative cells, K76AL/pHhyS-3 K-2α, were isolated on YPD + G418 plates.

**Fig. 4:**
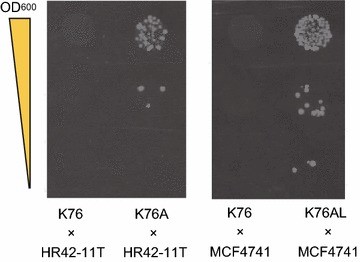
Evaluation of mating abilities of the derivative strains K76A and K76AL. Images showing colony formation on SD/MSG + G418 plates in the mating assay are shown. The Parent strains were K76 (**a**/α-type cells), and the Derivative strains were K76A (isolated as **a**-type cells) and K76AL (isolated as α-type cells), respectively. The OD_600_ values of 10-μL cell suspensions spotted on the solid media were as in Fig. 3b

For determination of mating type, all plasmids were removed via cultivation in YPD medium to yield strains K76A and K76AL (Table 1). In a mating assay, strain K76A successfully mated with HR42-11T (α-type) and K76AL mated with MCF4741 (a-type) (Fig. 4). These results suggest that **a-** and α-type derivative cells can be generated from hybrid strains (polyploids) as well as parental diploid strains. Notably, the generated K76A/pLhyS-2 K-Pa1and K76AL/pHhyS-3 K-2α derivative cells can be directly utilized for hybridization during continuous crossbreeding without removing plasmids pLhyS-2 K-Pa1and pHhyS-3 K-2α, respectively.

To verify the feasibility of performing continuous crossbreeding using the **a-** and α-type derivative cells, multi-hybridization was performed with K76A/pLhyS-2 K-Pa1 and K9AL/pHhyS-3 K-2α cells (isolated as described above) according to the scheme illustrated in Fig. 2b. The plasmid pAUR112 was introduced into strain K9AL after removing the plasmid pHhyS-3 K-2α, yielding K9AL/pAUR112 cells after selection on YPD + AUR plates. Mixed cultures of K76A/pLhyS-2 K-Pa1 and K9AL/pAUR112 were plated on YPD + HYG, AUR medium to select for hybrid K76x9/pLhyS-2 K-Pa1/pAUR112 cells, and all plasmids were then removed from the hybrid cells via cultivation in YPD medium without antibiotics to yield multi-hybrid strain K76x9 (Table 1).

### Estimation of cellular DNA content

To further characterize the derivative and hybrid cells, microscopic observation of strains K7A, K6AL, K9AL, K76, K76A and K76x9 was performed using BY4742 and Kyokai No. 7 as control haploid and diploid strains, respectively (Additional file 1: Fig. S2). The diameter of a yeast cell increases with increasing amounts of nuclear DNA (Amodeo and Skotheim 2016). The cell size of hybrid strain K76 was much larger than that of strains K7A and K6AL, suggesting that the DNA content within strain K76 cells had increased. In contrast, the cell size of multi-hybrid strain K76x9 was clearly smaller than that of strains K76 and K76A, suggesting that a major loss of DNA occurred after the hybridization of K76A and K9AL.

**Fig. 5:**
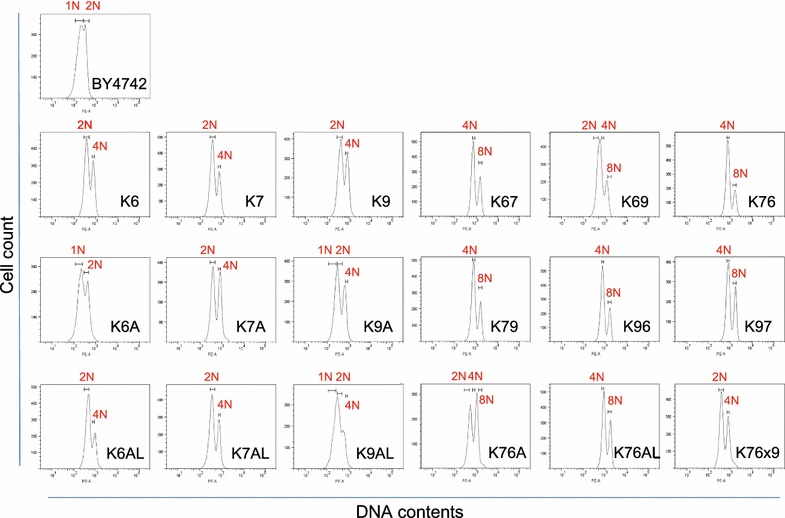
DNA content histogram of PI-stained yeast cells using FACS. PI-fluorescence data was collected from yeast cells within a gate drawn in the FSC-SSC* dot plots* (Additional file 1: Fig. S3) according to the data collected from control diploid strains. For relative comparison of DNA content, reference values of 2 N (two sets of all chromosomes) and 4 N (four sets of all chromosomes) were defined as the average of each peak value of Kyokai No.6, No.7 and No.9. The 1 and 8 N values were calculated from the 2 and 4 N values

To more accurately estimate the DNA content of cells, FACS analysis was performed after propidium iodide (PI)-staining of each yeast strain (Fig. 5). In DNA content histograms, two PI-fluorescence signal peaks, corresponding to the G0/G1 and G2/M phases, are typically observed (Qiu et al. 2013). The DNA content of diploid strains Kyokai No. 6, No. 7 and No. 9 was measured, and reference values of 2 N (two sets of all chromosomes) and 4 N (four sets of all chromosomes) were defined as the average of each peak value. The 3 N value was calculated as the average of the 2 and 4 N values, and 1, 1.5, 2.5, 4.5, 5, 6, 8 and 9 N values were then calculated from the 3 N value (Table 3). Notably, although haploid strain BY4742 is a laboratory yeast strain, the two peak values obtained for this strain were well accorded with the calculated 1 N and averaged 2 N value.

**Table 3:** Reference values used in the FACS analysis

Peak	Value of PI-fluorescence
1 N	189.18
1.5 N	283.77
2 N	379.28 ± 18.59
2.5 N	472.95
3 N	567.54
4 N	755.79 ± 35.58
4.5 N	851.30
5 N	945.89
6 N	1135.07
8 N	1513.43
9 N	1702.61

±means standard deviations from three diploid strains

The DNA content of sake yeast strains generated in the present study was measured and evaluated based on comparison to the reference values (from 1 to 9 N) (Table 4; Fig. 5). The results of FACS analysis (Additional file 1: Fig. S3) and microscopic observation (Additional file 1: Fig. S4) indicate that generated strains have cell flocculation ability, unlike the control strains. To exclude signals originating from the flocculated cells, a data collection gate was drawn according to FSC-SSC dot plots from the control strains (Additional file 1: Fig. S3). As shown in Table 4 and Fig. 5, strains K7A and K7AL had similar DNA contents to those of the parental strain (Kyokai No. 7). In contrast, strains K6A, K9A and K9AL appeared to have partially lost chromosomal DNA due to unequal distribution of DNA during mitotic cell division, whereas strain K6AL acquired additional chromosomal DNA.

**Table 4:** Evaluation of DNA content and ploidy assessment by FACS analysis

Strain	Peak 1	Peak 2	Ploidy
*Control*			
BY4742	188.34	345.07	1 N
Kyokai No.6	366.04	730.97	2 N
Kyokai No.7	366.22	730.30	2 N
Kyokai No.9	405.57	806.11	2 N
*Tested*			
K6A	207.37	413.17	1 N
K7A	379.61	806.97	2 N
K9A	280.76	616.19	1.5 N
K6AL	433.52	865.21	2.5 N
K7AL	331.98	728.50	2 N
K9AL	280.73	520.07	1.5 N
K67	717.23	1475.10	4 N
K69	547.61	1125.63	3 N
K76	718.22	1527.81	4 N
K79	739.99	1528.83	4 N
K96	717.00	1532.82	4 N
K97	766.30	1637.93	4 N
K76A	529.92	1022.16	3 N
K76AL	821.08	1640.31	4.5 N
K76x9	367.34	756.46	2 N

In the outcrossing experiments, the DNA content of hybrid strains K67, K69, K76, K79, K96 and K97 was found to be X ± 0.5 N, where X is the total DNA content of the two parental strains. However, strain K76A appeared to have lost an amount of DNA that was nearly equivalent to one set of chromosomes, whereas strain K76AL acquired additional DNA that was almost equivalent to a half set of chromosomes. It was also determined that the multi-hybrid strain K76x9 lost a substantial amount of chromosomal DNA during the mating process and maintained a near-diploid DNA content. These results are consistent with the microscopic observations of cellular morphology described above (Additional file 1: Fig. S2).

## Discussion

The aim of this study was to establish a versatile method for the crossbreeding of sake yeasts, which have low sporulation rates. We constructed two new plasmids for the isolation of **a**- and α-type derivative cells from **a**/α-type parental cells. The constructed plasmids required at least two types of marker genes: one for the selection of yeast transformants and one for the selection of **a-** or α-type derivative cells. To this end, we employed two commonly used marker genes for industrial yeast strains, namely the *hygro* and *kanMX4* genes, for the transformation and isolation of **a**- and α-type derivative cells, respectively (Murakami et al. 2012).

As **a**- and α-type yeast cells can be generated by spontaneous chromosomal mutations, such as LOH in the region containing the *MAT* locus or the mitotic loss of chromosome III (Alvaro et al. 2006; Andersen et al. 2008; Daigaku et al. 2004; Mayer and Aguilera 1990; Takagi et al. 2008), we examined the nuclear DNA of the generated **a**- or α-type derivative strains. The DNA content of cells after the removal of all plasmid DNA was evaluated qualitatively by the microscopic analysis of cell size and quantitatively by the FACS measurement of DNA content based on PI fluorescence signal intensity. In cases of derivative strains K6A, K9A, K9AL and K76A, the DNA content was clearly smaller compared to those of the parental strains, suggesting that the loss of chromosomal DNA occurred. In contrast, other derivative strains had DNA contents that were nearly equivalent to those of the parental strains. Notably, however, it is not possible to determine the rearrangement event that occurred in the obtained derivative strains based on these results alone, because the *MAT* locus only occupies 2 % of all chromosomes (Kumaran et al. 2013). Thus, although these methods do not require complicated or time-consuming procedures, estimation of chromosome III number within cells by real-time PCR (Fukuda et al. 2013a), array comparative genomic hybridization (Abunimer et al. 2016), or whole-genome sequencing would be needed to investigate the underlying events in more detail.

Although it was demonstrated that the basic method developed here (Fig. 2a) was applicable for the isolation of yeast cells with mating ability from common sake yeast strains, due to the requirement for the repeated exchange of plasmids, this approach still required multiple steps to perform the final hybridization and isolated **a**- or α-type derivatives. To reduce the need for plasmid exchange steps, we introduced a third marker gene, which was compatible with the *hygro* and *kanMX4* marker genes, into the selection system. Using the commercial plasmid pAUR112, which contains the *AUR1*-*C* marker gene, we successfully isolated hybrid cells without the need for the time-consuming plasmid exchange process. In a back-crossing comprising *n* cycles of hybridization, only a single plasmid exchange step would be required in the scheme illustrated in Fig. 2b and c, whereas (2*n* + 1) plasmid exchange steps would be needed in the scheme described in Fig. 2a.

To demonstrate the feasibility of the continuous crossbreeding approach developed in the present work, we generated a multi-hybrid strain, K76x9, from strains K76A and K9AL, with the expectation that the obtained strain would inherit genetic characteristics from strains Kyokai No. 6, No. 7 and No. 9. Microscopic observation and FACS analysis revealed that both strains K76A and K9AL underwent partial loss of chromosomal DNA, whereas the multi-hybrid strain K76x9 possessed a near-diploid DNA content, having lost a major part of its DNA during the mating process. There are two possible factors that lead to decreased genomic stability in the generated strains. First, aneuploidy (a chromosomal content differing from multiple sets of haploid chromosomes) induces genomic instability (Skoneczna et al. 2015). Strain K76x9 was generated from aneuploid strain K9AL and suspected aneuploid strain K76A, which appeared to have lost DNA), and hence, the zygotes must also be aneuploid, which might induce chromosomal loss. The other possible factor leading to genomic instability is an increase in the ploidy of yeast cells. According to a previous report (Kumaran et al. 2013), chromosomal stability is reduced as the ploidy of a cell increases. These two factors presumably had synergistics effects on the genomic instability of the multi-hybrid strain K76x9.

In general, crossbreeding induces genomic shuffling based on mechanisms of sexual reproduction (Snoek et al. 2015), and the resulting derivative strains may therefore acquire phenotypes different from those of the parent strains. In the present approach, LOH and the mitotic loss of chromosomal DNA occur randomly, and the generated hybrid yeast strains may undergo additional chromosomal loss after mating to reach stable genomic states. Such changes in ploidy are termed meiosis-like adaptation (Storchova 2014) and can result in the formation of derivative and hybrid cells with diverse genotypes and phenotypes, even from the same parental polyploid strain. The genomic instability of polyploids is beneficial for crossbreeding because it has contributed to the adaptation and evolution of yeasts in nature (Snoek et al. 2015).

In conclusion, we have established a complete procedure for the crossbreeding of industrially used sake yeasts possessing sporulation defects. The outcrossing of sake yeasts was achieved in all examined combinations, and the feasibility of continuous crossbreeding was demonstrated by generating a multi-hybrid strain. The method developed here may allow the numerous and valuable yeast resources, including sake yeasts, to be efficiently used for generation of new strains with desirable properties for industrial applications.

## Additional file

10.1186/s13568-016-0216-x Supporting Information for Materials and Methods.
