# Supplementary material for: Continuous crossbreeding of sake yeasts using growth selection systems for a-type and α-type cells
Source: AMB Express. 2016 Jul 8;6:45. doi: 10.1186/s13568-016-0216-x (PMC4938812; doi:10.1186/s13568-016-0216-x)
Supplement: Supplementary file 1 — 10.1186/s13568-016-0216-x Supporting Information for Materials and Methods. [file 13568_2016_216_MOESM1_ESM.pdf]

# Supplementary Materials

## *AMB Express*

## Continuous crossbreeding of sake yeasts using growth selection systems for a-type and α-type cells

Nobuo Fukuda[1]*, Misato Kaishima[2], Jun Ishii[3], Akihiko Kondo[2] and Shinya Honda[1]

[1]Biomedical Research Institute, National Institute of Advanced Industrial Science and Technology (AIST), Higashi, Tsukuba, Ibaraki 305-8566, Japan

[2]Department of Chemical Science and Engineering, Graduate School of Engineering, Kobe University, 1-1 Rokkodai, Nada, Kobe, Japan.

[3]Organization of Advanced Science and Technology, Kobe University, 1-1 Rokkodai, Nada, Kobe, Japan.

*Corresponding author

Fax: +81 29 861 6194

Tel: +81 29 849 1458

E-mail: nob-fukuda@aist.go.jp

## Doc. S1

## Supporting Information for Materials and Methods

**PCR assay to confirm removal of unnecessary plasmids.** The oligonucleotides used for this assay are listed in Table S3. The plasmids pLhyS-2K-Pa1 and pHhyS-3K-2α contain *kanMX4* and *hygro* selection markers as exogenous genes. To confirm removal of these plasmids, the hygro gene was amplified from the genomic DNA derived from each yeast strain with the oligonucleotide pair o1 and o2, and the kanMX4 gene was amplified with the oligonucleotide pairs o3 and o4 (for pLhyS-2K-Pa1) or o5 and o4 (for pHhyS-3K-2α). The amplified DNA fragments were visualized by agarose gel electrophoresis.

**Microscopic observation.** Each yeast strain was cultured overnight in 500 μL YPD medium at 30°C. The cells then were harvested, washed with 500 μL distilled water, and resuspended in 100 μL phosphate buffered saline (PBS) containing 4 μM Hoechst 33342 (Dojindo Laboratories Co., Ltd, Kumamoto, Japan) to stain nuclear DNA within yeast cells. After 1 h of incubation at room temperature, each cell suspension was diluted 20 fold using PBS. Stained cells were observed using a fluorescent microscope (BZ-X700, Keyence Co., Ltd, Tokyo, Japan). Each image was photographed with the same exposure time using a 60x objective lens. Fluorescent images were overlaid on the corresponding bright-field images.

**Table S1.** Hygromycin B (HYG)-resistance of parental yeasts grown in YPD medium. The $OD_{600}$ values of the YPD cultures are presented.

| | Concentration of HYG [μg/ml] | | | | |
|---|---|---|---|---|---|
| Strain 0 | 100 | 200 | 300 | 500 | |
| K6 | 6.55 | 5.46 | 4.00 | 0.09 | 0.02 |
| K7 | 6.79 | 3.18 | 0.10 | 0.08 | 0.10 |
| K9 | 6.88 | 2.79 | 0.22 | 0.10 | 0.11 |

**Table S2.** Geneticin (G418)-resistance of parental yeasts grown in YPD medium. The $OD_{600}$ values of the YPD cultures are presented.

| | Concentration of G418 [μg/ml] | | | |
|---|---|---|---|---|
| Strain 0 | 100 | 200 | 300 | |
| K6 | 7.38 | 0.48 | 0.13 | 0.11 |
| K7 | 7.01 | 0.24 | 0.11 | 0.11 |
| K9 | 7.09 | 0.18 | 0.10 | 0.10 |

**Table S3.** Sequences of oligonucleotides used for PCR assay.

| Number | Sequence |
|---|---|
| 1 | 5'-CAAAgcggccgcATGGATAGATCCGGAAAGCC-3' |
| 2 | 5'-AATTTATTTCggatccCTATTCCTTTGCCCTCGGAC-3' |
| 3 | 5'-GAATCAAAAgcggccgcATGGGTAAGGAAAAGACT-3' |
| 4 | 5'-CCCCAGTTTGggatccTTAGAAAAACTCATCGAGC-3' |
| 5 | 5'-AAAATTTTCgcggccgcATGGGTAAGGAAAAGACT-3' |

**Table S4.** Aureobasidin A (AUR)-resistance of K7A and K6AL grown in YPD medium. The $OD_{600}$ values of the YPD cultures are presented.

| | Concentration of AUR [ng/ml] | | | |
|---|---|---|---|---|
| Strain 0 | 100 | 300 | 500 | |
| K7A | 1.82 | 0.14 | 0.08 | 0.04 |
| K6AL | 2.83 | 0.32 | 0.08 | 0.09 |

## Supplementary Figures

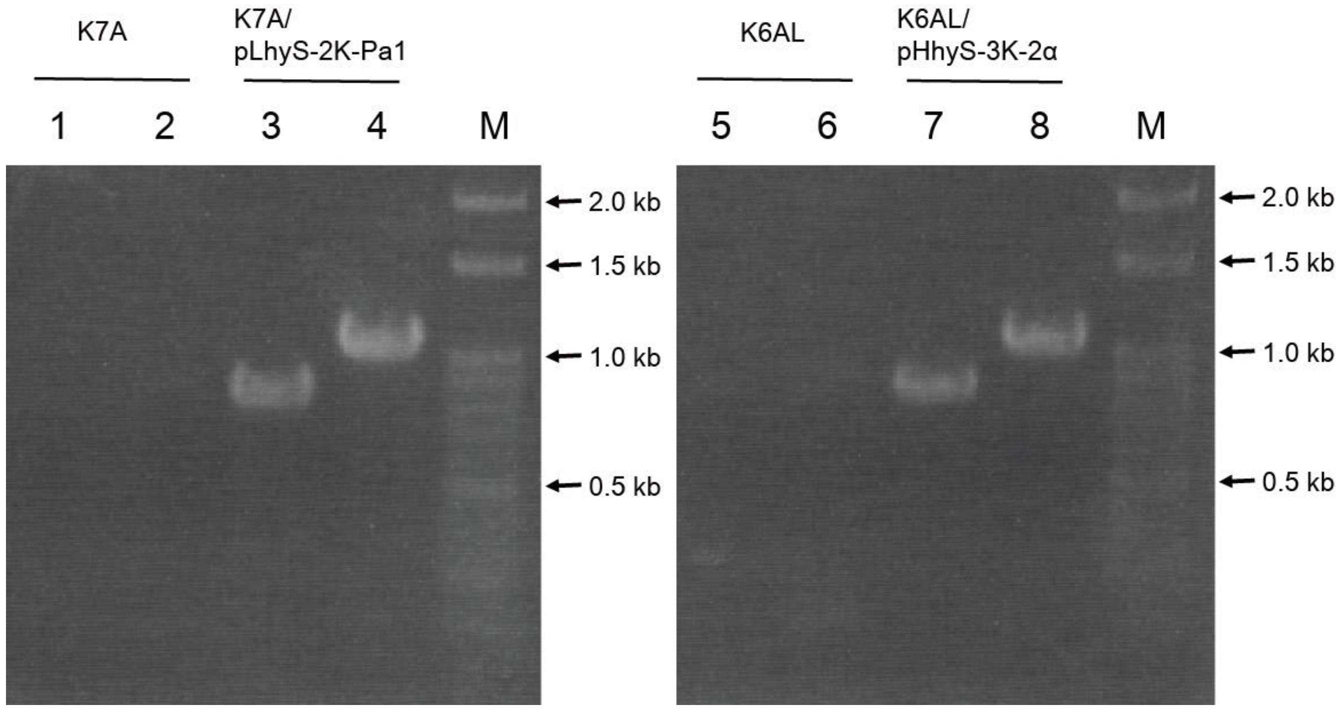


**Figure S1. Confirmation of removal of unnecessary plasmids.** The fragment size of the *kanMX4* gene (ORF) is 810 bp, and that of the *hygro* gene (ORF) is 1,035 bp. The target region for amplification in each lane is as follows: Lanes 1, 3, 5, and 7: *kanMX4*. Lanes 2, 4, 6, and 8: *hygro*. Lane M: DNA size marker.

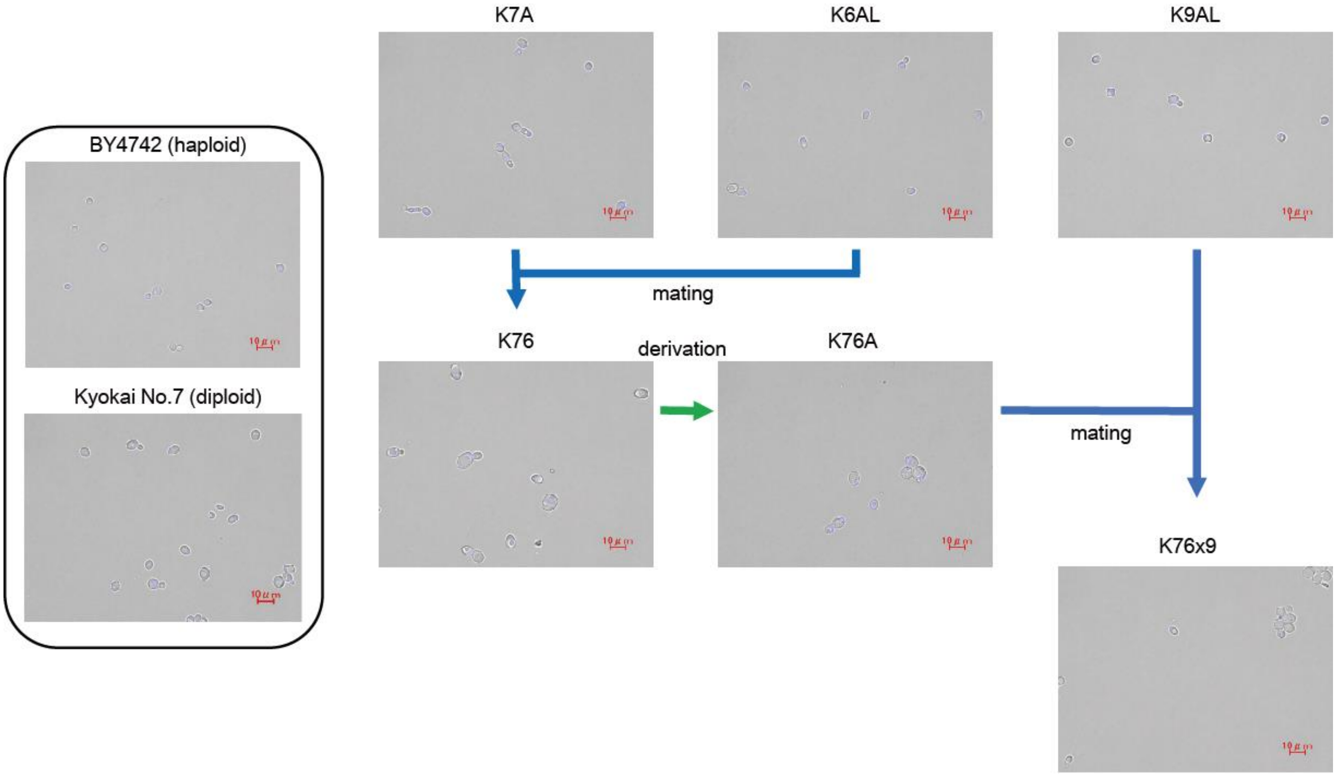


**Figure S2. Observation of cellular size during multi-hybridization.** Nuclear DNA was stained with Hoechst 33342. Scale bar: 10 µm.

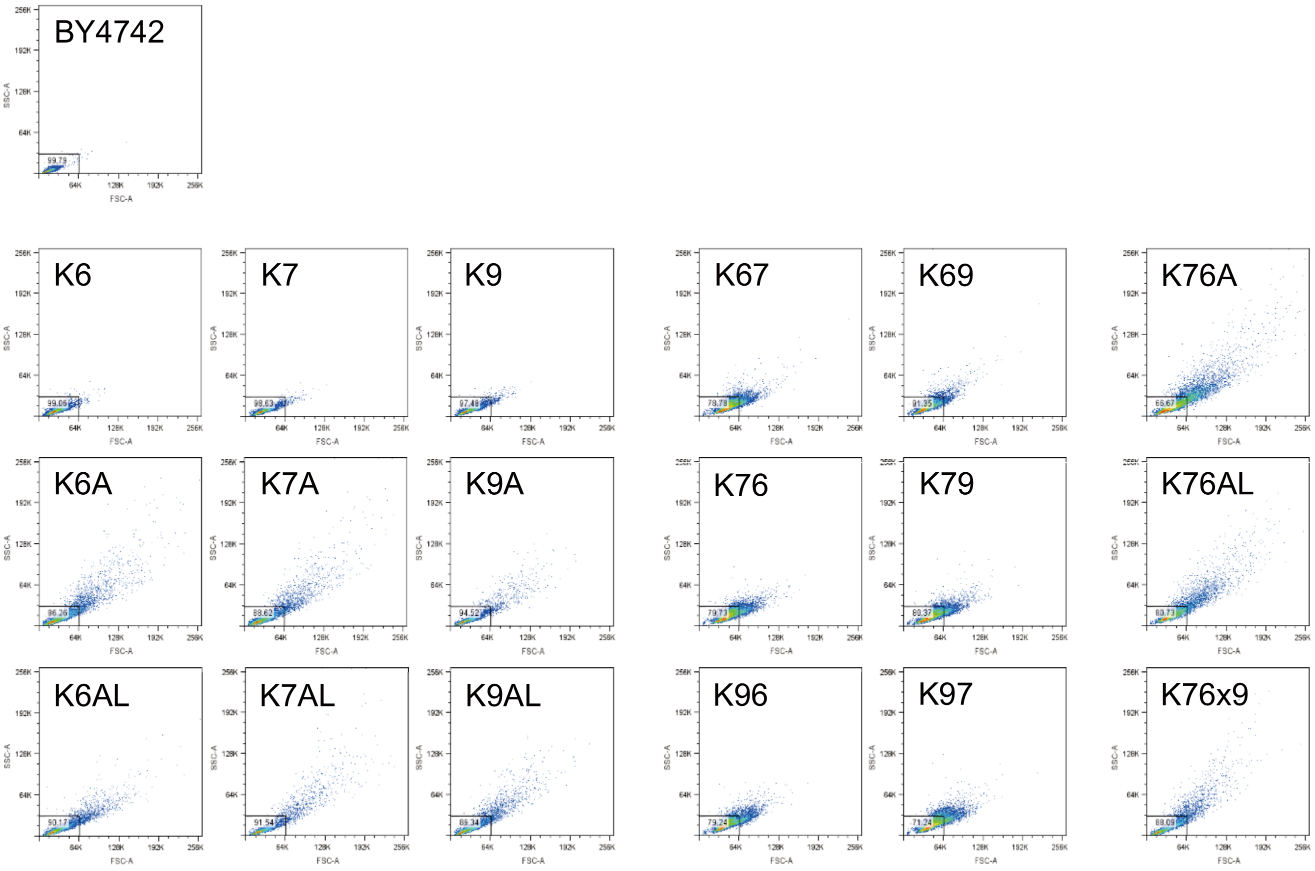


**Figure S3. FSC-SSC dot plots of particles detected by FACS.** A data collection gate was set according to non-flocculated control strains (haploid and diploid) to exclude signals emitted from flocculated cells.

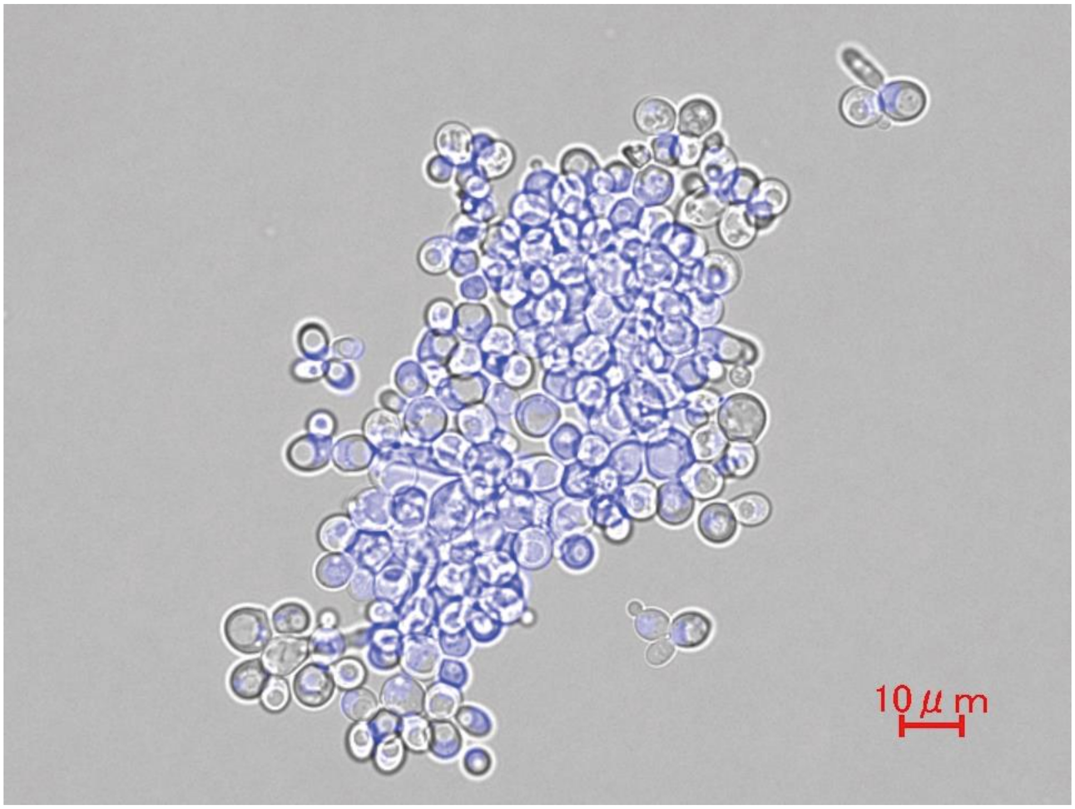


**Figure S4. Cell flocculation of the mating-type-converted strain K7A.** Nuclear DNA was stained with Hoechst 33342. Scale bar: 10 μm.
